# *In Situ* Formation of Nanoporous Silicon on a Silicon Wafer via the Magnesiothermic Reduction Reaction (MRR) of Diatomaceous Earth

**DOI:** 10.3390/nano10040601

**Published:** 2020-03-25

**Authors:** Patrick Aggrey, Bakhodur Abdusatorov, Yuliya Kan, Igor A. Salimon, Svetlana A. Lipovskikh, Sergey Luchkin, Denis M. Zhigunov, Alexey I. Salimon, Alexander M. Korsunsky

**Affiliations:** 1 Hierarchically Structured Materials lab, Center for Energy Science and Technology, Skolkovo Institute of Science and Technology, 121205 Moscow, Russia; patrick.aggrey@skoltech.ru (P.A.); bakhodur.abdusatorov@skoltech.ru (B.A.); Yuliya.Kan@skoltech.ru (Y.K.); s.lipovskikh@skoltech.ru (S.A.L.); s.luchkin@skoltech.ru (S.L.); a.salimon@skoltech.ru (A.I.S.); 2 Center Photonics and Quantum Materials, Skolkovo Institute of Science and Technology, 121205 Moscow, Russia; igor.salimon@skoltech.ru (I.A.S.); D.Zhigunov@skoltech.ru (D.M.Z.); 3 Multi-Beam Laboratory for Engineering Microscopy (MBLEM), Department of Engineering Science, University of Oxford, Oxford OX1 3PJ, UK

**Keywords:** magnesiothermic reduction reaction, diatomaceous earth, *in situ* processing, nanoflakes, black silicon, fractal structure, surface reflection, light absorption

## Abstract

Successful direct route production of silicon nanostructures from diatomaceous earth (DE) on a single crystalline silicon wafer via the magnesiothermic reduction reaction is reported. The formed porous coating of 6 µm overall thickness contains silicon as the majority phase along with minor traces of Mg, as evident from SEM-EDS and the Focused Ion Beam (FIB) analysis. Raman peaks of silicon at 519 cm^−1^ and 925 cm^−1^ were found in both the film and wafer substrate, and significant intensity variation was observed, consistent with the SEM observation of the directly formed silicon nanoflake layer. Microstructural analysis of the flakes reveals the presence of pores and cavities partially retained from the precursor diatomite powder. A considerable reduction in surface reflectivity was observed for the silicon nanoflakes, from 45% for silicon wafer to below 15%. The results open possibilities for producing nanostructured silicon with a vast range of functionalities.

## 1. Introduction

Facile fabrication of nano- or microporous silicon surface has been pursued over many years for potential applications in solar cells [1], lithium-ion batteries [2,3], microelectromechanical systems [4], H_2_ production by photo-electrochemical splitting of water [5], drug delivery [6], optoelectronic and photonic devices [7,8,9,10,11,12,13,14,15], and chemical and biological sensors [16,17,18,19,20]. Bulk silicon has found applications in photodiodes, photodetectors, and photovoltaic devices [7]. However, there are limitations with the direct use of bulk silicon especially in photovoltaic devices, due to its high surface reflectivity and large bandgap. Surface modifications through wet etching have been reported to produce porous silicon surfaces [21]. Black silicon (BSi), a nanostructured silicon wafer surface, has gained a lot of attention in this area of research due to the reports of suppressed reflection and simultaneous enhancement of scattering and absorption of light [1,22,23]. This silicon type appears black, in contrast with the silver-grey color of planar silicon wafers. Black silicon has been produced through techniques ranging from HF etching, stain etching, metal assisted chemical etching, laser irradiation, and the molten salt Fray–Farthing–Chen Cambridge (FFC Cambridge) process [1]. Although, these techniques give positive results, a number of drawbacks have also been reported. In the case of electrochemical etching, there is difficulty applying this technique to a larger wafer surface area. A possible metal contamination and therefore subsequent thorough metal removal is required in the case of the metal-assisted chemical etching. Additionally, in both reactive-ion etching and laser treatment, a considerable amount of damage is made to the silicon substrate. Reactive-ion etching also comes with a high cost of production. Lastly, the FFC Cambridge process requires a relatively high temperature and renders the wafer prone to contamination by Mo and other metals. Almost all the techniques presented in the literature to date require the removal of the top surface of the silicon wafer.

Metallothermic reduction reaction (MRR) of silica to silicon has been studied over the past decade [24,25,26,27,28]. This technique has gained a lot of attention because of the low cost involved, lower temperature, and the ready availability of metals such as magnesium and aluminum. Since Sandhage et al. reported the conversion of diatom frustules to porous silicon replicas using magnesium vapor [27], significant further progress has been made in the area [24,25,26,28]. To the best of our knowledge, the majority of these reports have focused on producing silicon powders. For a nanoporous silicon surface, precursor silica with inherent nanoporous structure is desirable. Diatomaceous earth (DE), a naturally occurring and eco-friendly material, is a siliceous sedimentary rock formed from fossilized remains of diatom frustules [29]. This makes diatomite a suitable silica precursor for the production of nanoporous silicon structures on silicon wafer surfaces.

In this paper, we focused on the direct formation of nanoporous silicon adherent layer on silicon wafer substrate through a direct reduction of reaction mixture (SiO_2_ + NaCl + Mg) on a silicon wafer substrate. Abundant diatomite sources offer a unique opportunity to produce nanoporous silicon structures on a silicon wafer substrate with neat naturally formed nanoporous structure. Magnesium is readily available as the reducing agent for the reduction process. The entire reaction mixture, before and after reduction, is not as environmentally harmful as in the case of *SF*_6_ used in the some of the reported techniques for the formation of black silicon. The magnesiothermic reduction reaction also offers a fairly simple set-up and a lower processing temperature compared to the conventional method of producing silicon, carbothermal reduction, for which processing temperatures of 2000–2200 °C are required. Since these high temperatures are not favorable for the preservation of the nanoporous structure of the precursor diatomite powder, an alternative lower temperature route must be established.

## 2. Materials and Methods 

### 2.1. Sample Preparation

The diatomite powder used was purchased from QUANTUM Ltd., Nikolsk (Penza, Russia) with the chemical composition shown in Table 1. The as-supplied diatomite powder was well mixed with NaCl that was used as heat scavenger of the high energy output of the exothermic metallothermic reaction, as reported by Luo et al. [28]. The reduction protocol was applied as follows:

- Reduction temperature of 700 °C for 2.5 h;
- The molar ratio of SiO_2_ and Mg was 1:1.25.

The processing steps were taken as follows:

- Diatomite earth was mixed with aqueous NaCl solution in the weight ratio 1:10. The mixture was well stirred using a magnetic stirrer at 70 °C.
- The solution was centrifuged and the slurry dried at 85 °C overnight.
- The obtained powder was mixed with magnesium powder in the molar ratio of 1:1.25 with a pestle and mortar.

### 2.2. Powder Particle Size and Surface Area

The diameter and the particle size distribution of the samples were measured using Fritsch Particle Analyzer (FRITSCH, Idar-Oberstein, Germany). The pore size and specific surface area were determined using the Quantachrome Nova 2200e BET porosimeter (Anton Paar, Graz, Austria). Degassing was performed at 200 °C for 1000 min.

### 2.3. Synthesis of Silicon Nanoflakes on Silicon Wafer Substrate

**Figure 1:**
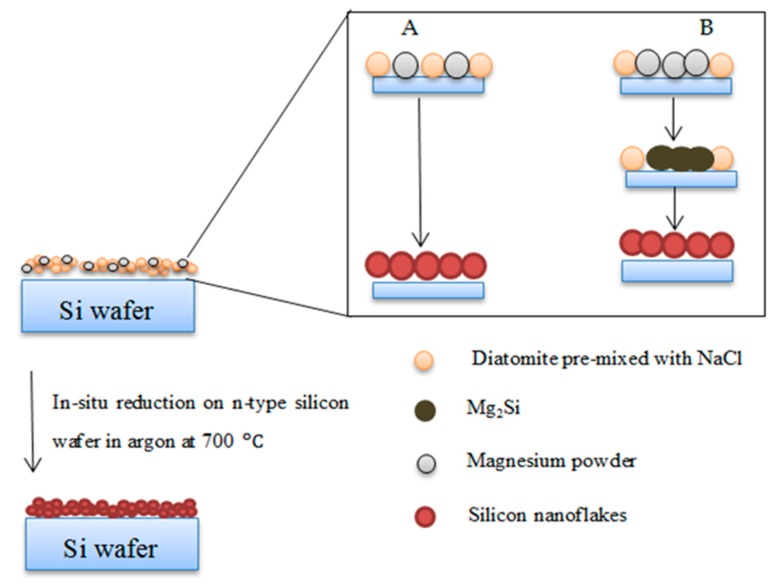
Schematic representation of strategy used to produce silicon nanoflakes. Zoom in: illustrating possible reaction routes at the silicon wafer surface.

The surface of an n-type silicon wafer with orientation (001) from the Terra Group OOO, Moscow, was modified using the thermite reduction of diatomite powder. The strategy employed is shown in Figure 1. A slurry of the reaction mixture (SiO_2_ + NaCl + Mg) was made in a petri dish using ethanol. To achieve homogeneity, the slurry was mixed several times. The slurry was quickly transferred to the silicon wafer surface with the help of a pipette and allowed to dry. The silicon wafer with the dried reaction mixture was reduced under the flow of argon in an electric furnace at 700 °C for 2.5 h. The silicon wafer surface was coated with coffee-brown nanoporous silicon clusters. The remaining loose powder after the thermite reaction was collected, etched with 1 M HCl and 5% HF, and rinsed in deionized water, to obtain silicon powder—the purification treatment (hereinafter designated as “as-etched”). The obtained powder was brownish-yellow in color, which is similar to colors reported in the literature for porous silicon [30]. The silicon wafer, after the in situ MRR, was purified in the same manner as the loose powder to wash out NaCl, MgO, and unreacted silica.

### 2.4. Microstructural Analysis

Microstructural analysis of raw and reduced diatomite powders was performed on the Helios dual beam system (Thermo Fisher Scientific, Waltham, MA, USA) that combines a scanning electron microscope with a Focused Ion Beam. The surface topography was observed, and Focused Ion Beam (FIB) cross-sectional milling was performed at the sample tilt angle of 52° after applying a protective platinum coating. Energy Dispersive X-ray spectroscopy (EDX) analysis was carried out to determine the elemental composition.

### 2.5. X-ray Diffraction Analysis

The phase composition of raw diatomite, as-reduced mixture and nanoporous silicon powder were investigated using the Bruker D8 Advance Diffractometer (Bruker, MA, USA) with Cu Kα_1_ radiation (*λ =* 1.5405980 *Å*). The broad peak confirms the amorphous nature of raw diatomite with a major amorphous phase being the biogenic opal A silica with mineral impurities such as quartz silt and sand, clay minerals, iron oxide, carbonate minerals, and organic matter [31].

### 2.6. Raman Spectroscopy

Raman spectra were collected at room temperature using a DXR2xi Raman Imaging Microscope (Thermo Fisher Scientific, MA, USA). The excitation source used was the 532 nm line of an Ar-ion laser. 

### 2.7. Optical Property Measurement

The spectrum was obtained using the Optical Spectrum Analyzer BOSA 440 (Aragon Photonics, Zaragoza, Spain) in the infrared (IR) region.

### 2.8. Atomic Force Microscopy

Kelvin Probe Force Microscopy (KPFM) measurements were performed in a 2-pass amplitude modulation mode with the 30 nm second pass height on Cypher ES Atomic Force Microscope (Oxford Instrument, Abingdon, UK) under dry Ar atmosphere (O_2_ < 0.1 ppm, H_2_O < 0.1 ppm) in dark and under illumination with a blue laser (*λ =* 405 nm). Si AFM probe with conductive W_2_C coating, 245 kHz first resonance frequency, and 9.4 N/m spring constant was used. The contact potential difference (*V_CPD_*) measured between the silicon nanoflakes/silicon wafer and the AFM probe was used to estimate samples’ work function.

## 3. Results

### 3.1. Powder Particle Size and Surface Area 

**Figure 2:**
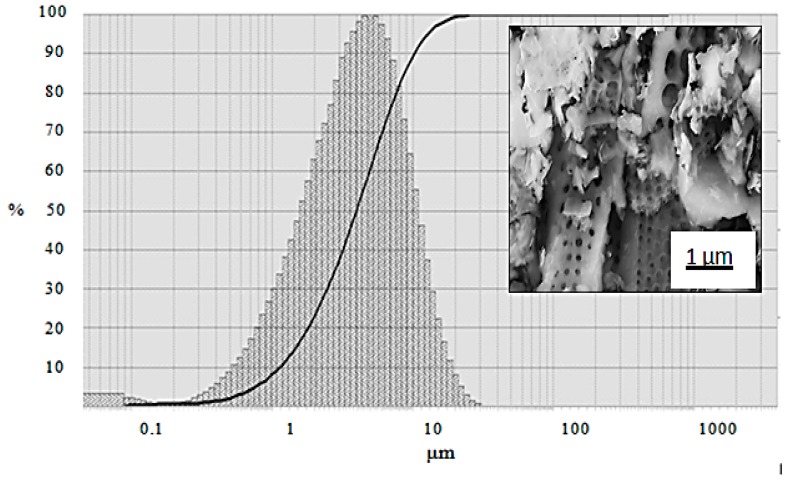
Powder particle size distribution of diatomite powder. Inset: SEM microstructural image of precursor diatomite powder showing crushed frustules with some porous structure.

The specific surface area of the as purchased diatomite powder was 20.4306 m^2^/g. The histogram of the particle size distribution of the diatomite powder is shown in Figure 2. The primary particle sizes in diatomite powder were in the range of 1–10 µm. For industrial applications, the as-purchased diatomite powder was preprocessed at 900 °C. Such a heat treatment, often referred to as calcination, can lead to phase transformations, a reduction in surface area, and an increase in mean particle size and increase in hardness. Calcined diatomite is widely used in filters [31]. Both calcined and non-calcined diatomite precursors have been used in the MRR of diatomite [24,27]. In this study, no further heat treatment was carried out before mixing with NaCl and Mg. 

### 3.2. Phase Composition of the Reaction Mixture

**Figure 3:**
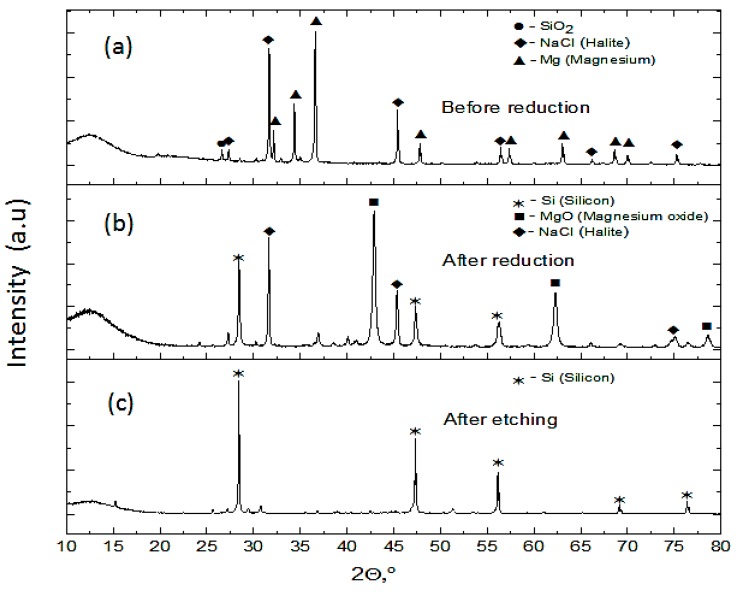
XRD pattern: (**a**) reaction mixture before the metallothermic reduction reaction (MRR), (**b**) remaining loose powder after the MRR, and (**c**) silicon obtained from remaining loose powder after etching.

**Figure 4:**
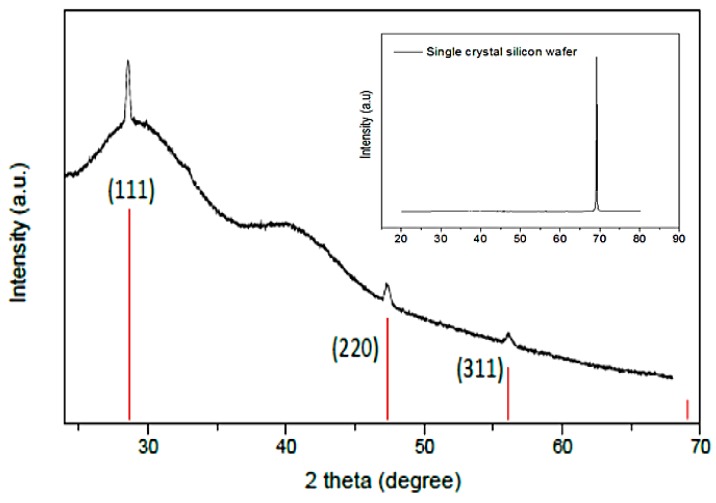
XRD pattern of the as-etched silicon nanoflakes. Inset: XRD pattern of the n-type silicon wafer with orientation (001).

The XRD patterns of all samples before the MRR show peaks of NaCl, SiO_2_, and Mg. In Figure 3a, the diffraction pattern of the reaction mixture is shown. The broad background in the diffraction pattern shown in Figure 3a was observed from the contribution of opal. This confirms the amorphous nature of raw diatomite. In addition to opal, there is some amount of crystalline quartz in raw diatomite [32]. Mineral impurities including quartz, clay minerals, iron oxide, carbonate minerals, and organic matter are known to influence the specific gravity (SG) and bulk density of diatomite. They increase both specific gravity and bulk density while reducing the porosity of diatomite [31]. For a highly nanoporous silicon product, a very pure precursor diatomite is needed. The remaining loose powder after the thermite reduction contained Si, MgO, NaCl, and unreacted SiO_2_ as seen in the diffraction pattern in Figure 3b. Further etching of the loose powder with 1 M HCl, 5% HF, and deionized water resulted in silicon powder with minor traces of opal as shown in Figure 3c. In Figure 4, the diffraction pattern of the as-purified silicon nanoflakes clusters formed directly on the single crystalline silicon wafer substrate is shown. Very intense diffraction peak of a single crystalline silicon wafer will be observed if all Bragg conditions were fulfilled. From Figure 4 however, no contribution from the substrate was seen. The diffraction peaks of silicon were observed, however, characterized by high backgrounds and low intensity peaks associated with nanocrystalline and/or amorphous nature of the silicon nanoflakes. 

A number of possible unwanted reactions accompanying MRR have been reported in the literature [26]. In this work, these obstacles were successfully overcome as shown in results from XRD. The formation of possible by-products, such as Mg_2_Si and Mg_2_SiO_4_, was not observed in the diffraction pattern. Increasing the Mg stoichiometric ratio is known to decrease the silicon yield while facilitating the formation of Mg_2_Si. On the other hand, a very low amount of Mg powder led to insufficient Mg at the Mg/SiO_2_, which also favors the formation of Mg_2_SiO_4_ [33]. These byproducts directly affect the silicon yield and the quality of the product silicon. Mg_2_SiO_4_ cannot be easily etched with HCl and therefore can affect the quality of the product silicon [26]. 

### 3.3. Microstructure of Silicon Nanoflakes

**Figure 5:**
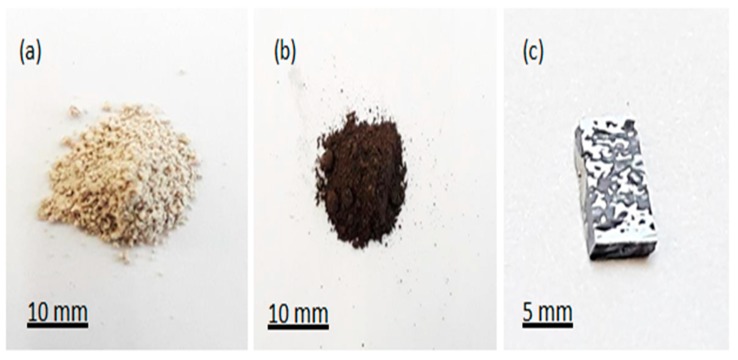
Photograph (**a**) reaction mixture before reduction, (**b**) remaining loose powder after reduction, and (**c**) silicon wafer surface after the reduction and etching.

**Figure 6:**
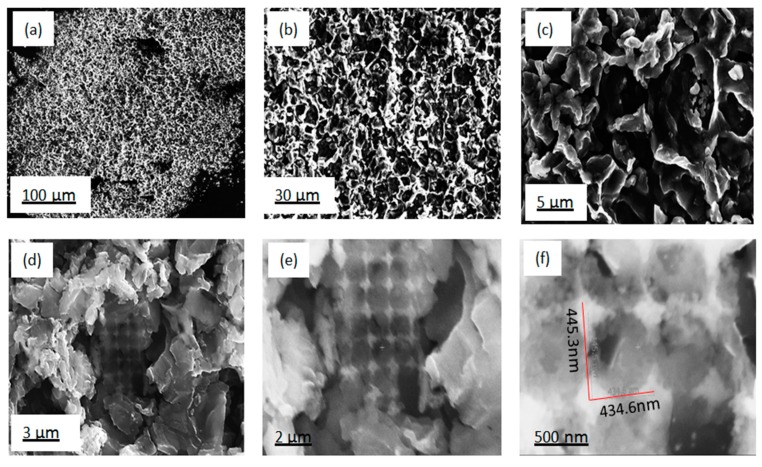
SEM images of the as-etched Si nanoflakes: (**a)** very low magnification surface view, (**b**) low magnification surface view, (**c**) large magnification surface view, (**d**) specific highly ordered feature, (**e**) large magnification of a specific highly ordered feature, and (**f**) nanoporous nature of a specific highly ordered feature.

**Figure 7:**
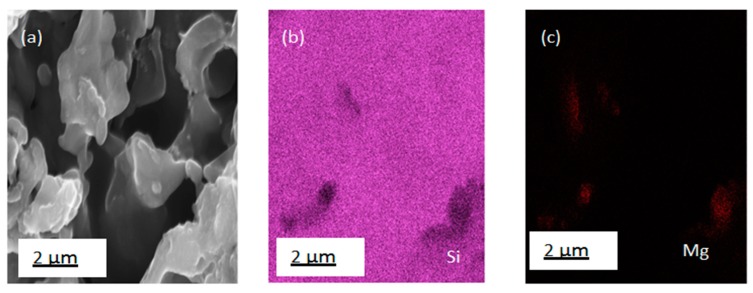
EDS elemental mapping of silicon nanoflakes after purification: etching with 1 M HCl, 5% HF and washing in deionized water. (**a**) SEM image of nanoflakes, (**b**) Si, and (**c**) Mg < 1% average.

A photograph of the silicon wafer after the surface modification is presented in Figure 5c. In Figure 5a,b, images of the reaction mixture before and after the reduction are shown. The microstructure of the precursor diatomite powder used is shown in Figure 2. Microstructural images of the Si nanoflakes produced directly from the reaction mixture via the magnesiothermic reduction on a silicon wafer are shown in Figure 6a–c. The microstructural images show fractal structures with some pores retained from the precursor diatomite instead of the peaks and valleys normally associated with BSi. In Figure 6d, a specific highly ordered feature is shown. The nanoporous nature of this ordered feature is shown in Figure 6e,f with a pore size of around 450 nm. Further EDS analysis of the microstructures shown in Figure 6 confirmed the presence of silicon with minor traces of magnesium as shown in Figure 7a–c. A number of reaction routes are possible in the direct formation of the silicon nanoflakes. In the MRR, the main reaction was between silica and magnesium as shown in Equation (1) and Figure 1. However, the possibility of forming Mg_2_Si between the silicon wafer and magnesium was also likely in this synthesis route. In the event of Mg_2_Si formation, a continuous reduction of the closest SiO_2_ particles by Mg_2_Si was likely to occur. Barreti et al. reports of a high yield in silicon achieved at a higher temperature with an Mg stoichiometric ratio increased above 2:1 [34]. The increased Mg stoichiometric ration favored the formation of Mg_2_Si. As a result, more silicon was produced through the solid state reduction of unreacted SiO_2_ by Mg_2_Si as shown in Equation (2). In this case however, an Mg: (SiO_2_ + NaCl) molar ratio of 1.25:1 shows that the possible Mg_2_Si formation was not due to a higher Mg stoichiometric ratio above 2:1. Rather, a direct contact of the wafer surface with any Mg particle might lead to the formation of Mg_2_Si as shown in Figure 1. The Mg_2_Si formed in such a case is then likely to reduce surrounding SiO_2_ particles as shown in Equation (2).

SiO_2_ + 2Mg → Si + 2MgO, (1)

SiO_2_ + Mg_2_Si → 2Si + 2MgO, (2)

### 3.4. FIB-SEM Cross-Section Imaging

**Figure 8:**
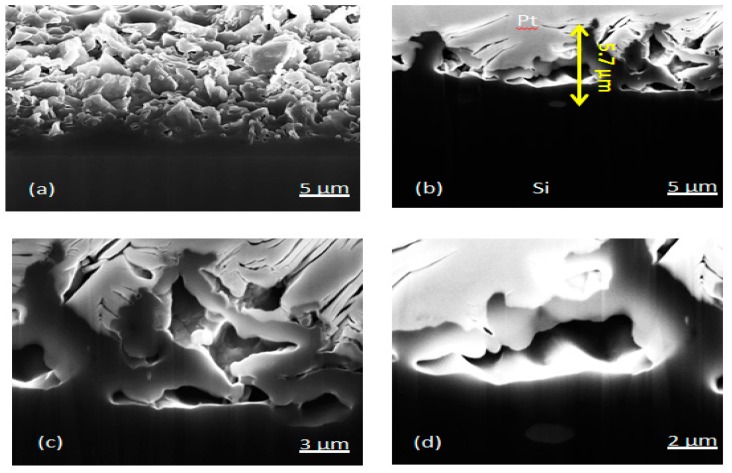
FIB-SEM cross-sectional images of the as-etched silicon nanoflakes: (**a**) low magnification without Pt coating, (**b**) low magnification with Pt coating, (**c**) large magnification showing micropores and cavities, and (**d**) magnified SEM image of cross-section.

The silicon nanoflakes were cross-sectioned using the Focused Ion Beam to confirm the *in situ* bonding of reduced diatomite powder to the silicon wafer surface. The thickness of the silicon nanoflakes as estimated from FIB analysis was 5.7 µm. Figure 8a–d shows microstructures of the cross-section of the in situ formed silicon nanoflakes. The cross-sectional view of the nanoflakes shows irregular peaks and valleys. Very few flakes possessed some nanoporous details from the precursor diatomite powder. On the contrary, there existed micropores and cavities between flakes of different layers and orientations.

**Figure 9:**
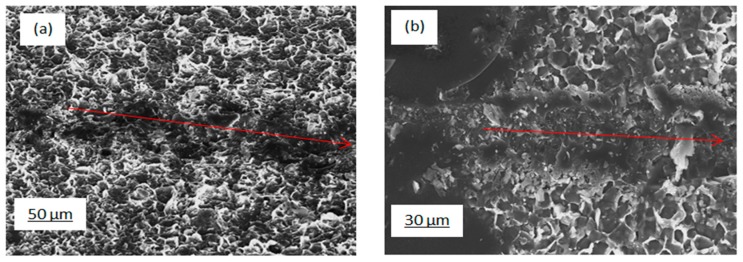
SEM images of the as-etched silicon nanoflakes: (**a**) scratch path through silicon nanoflakes at low magnification and (**b**) scratch path silicon nanoflakes at high magnification (red arrow showing scratch path).

From FIB-SEM, no clear contrast could be seen between the silicon nanoflakes and the wafer surface, suggesting an even bonding of the flakes to the silicon wafer. In Figure 9a,b, SEM images of the adhesion scratch test of the nanocrystalline Si coating on Si wafer substrate are presented. It is apparent from this qualitative assessment that good bonding was achieved, resulting in a ‘ploughing’ response without any flaking or spalling off of the coating.

### 3.5. Raman Scattering of Silicon Nanoflakes

**Figure 10:**
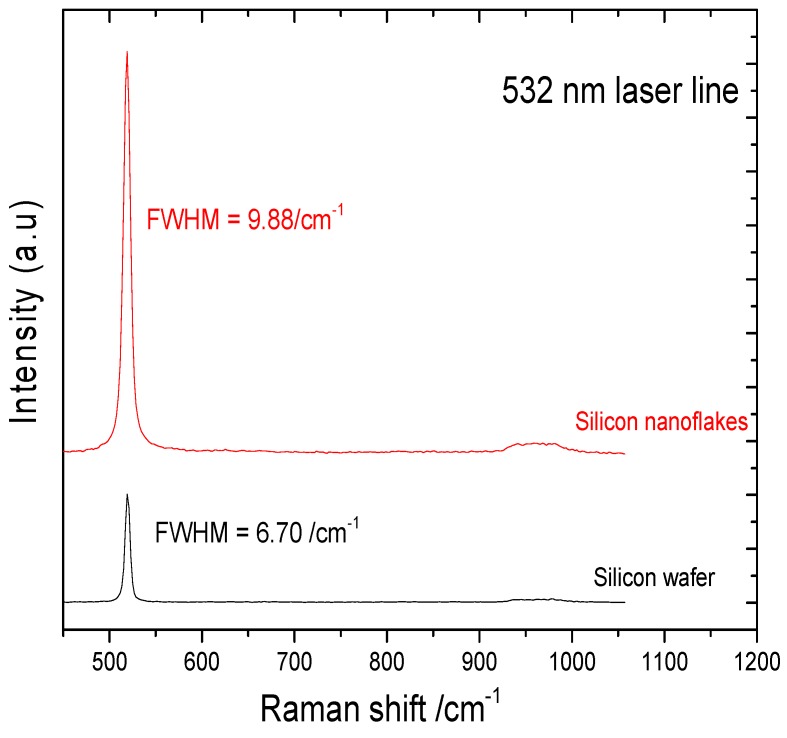
Raman spectra of silicon wafer and the as-etched silicon nanoflakes.

The Raman spectra of both monocrystalline silicon and nanoporous silicon are shown in Figure 10. Both first and second order scattering of silicon were observed in both spectra. However, the intensities of the peaks varied arbitrarily. The full width at half maximum (FWHM) of both detected peaks differed significantly. The FWHM values of the most intense peak at 519 cm^−1^ where 6.70 cm^−1^ for the substrate silicon wafer, and 9.88 cm^−1^ for the silicon nanoflakes. The level of disorder in materials was closely related to the FWHM. It has been reported by Casiraghi et al. [35] that FWHM always increases with disorder. The Raman intensity of the peaks from the nanoporous silicon was relatively higher than that of peaks from the bulk silicon. This could be related to the effects of nanolayers of ultrathin silicon flakes formed after the magnesiothermic reduction reaction. Raman intensities studies of MoS_2_ monolayers by Li et al. showed that intensities of the monolayers were relatively higher than the bulk MoS_2_ and varied arbitrarily according to the number of layers [36]. Additionally, Zuo et al. reported that, Raman enhancement was observed from conical cavity arrays [37]. They attributed this phenomenon to the significant Raman enhancement of molecules on the cavity walls. Cross-sectional analysis of the silicon nanoflakes via FIB milling showed the presence of pores as seen in Figure 8. These factors may account for the huge difference in intensities between silicon nanoflakes and the silicon wafer surface.

### 3.6. Optical Properties of Silicon Nanoflakes

**Figure 11:**
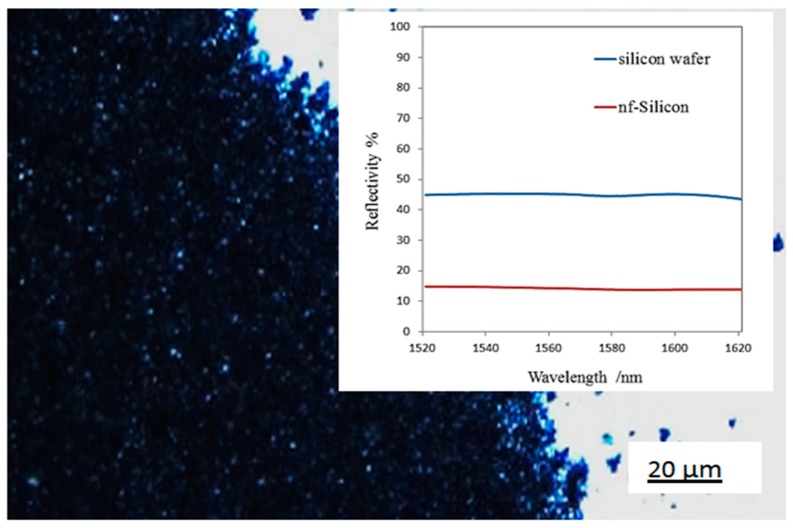
Optical microscope image of the as-etched silicon nanoflakes. Inset: reflectivity of silicon nanoflakes (nf-silicon; black) and silicon wafer surface (white surface) in the infrared region.

In Figure 11, an optical image of silicon nanoflakes after the reduction process is shown. The white background represents a bulk silicon surface while the black surface represents silicon nanoflakes. Whereas BSi appears black, the silicon nanoflakes appeared coffee brown in color. This is identical to the residual powder, which was easily brushed off as shown in Figure 5b. The reflectance spectrum of the silicon nanoflakes is presented in Figure 11. The silicon nanoflakes suppressed light reflection to less than 15% in the infrared region compared to the 45% light reflection from the monocrystalline silicon wafer surface. Like black silicon, silicon nanoflakes simultaneously enhanced light trapping. In the case of black silicon, the peaks and valleys are known to be responsible for the enhanced light absorption, as incident photons are reflected into the peaks and absorbed by the materials. The reflectivity of materials as reported by Stephens and Cody [38] are greatly reduced by light trapping through multiple reflections. With the Si nanoflakes, both irregular peaks and valleys and some pores contributed to the overall decrease in surface reflection of the silicon wafer.

### 3.7. AFM Measurement

**Figure 12:**
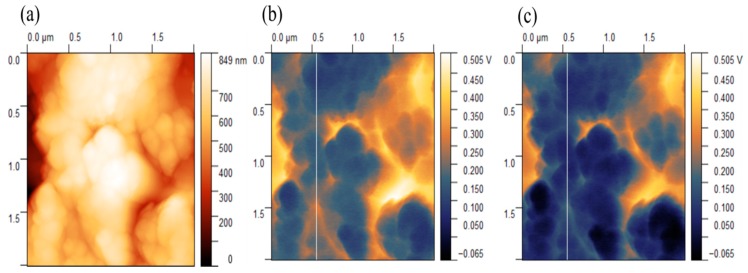
(**a**) Atomic Force Microscopy topography, (**b**) the Kelvin Probe Force Microscopy (KPFM) map of the as-etched silicon nanoflakes under dark conditions, and (**c**) KPFM map of silicon nanoflakes under the illumination condition.

**Figure 13:**
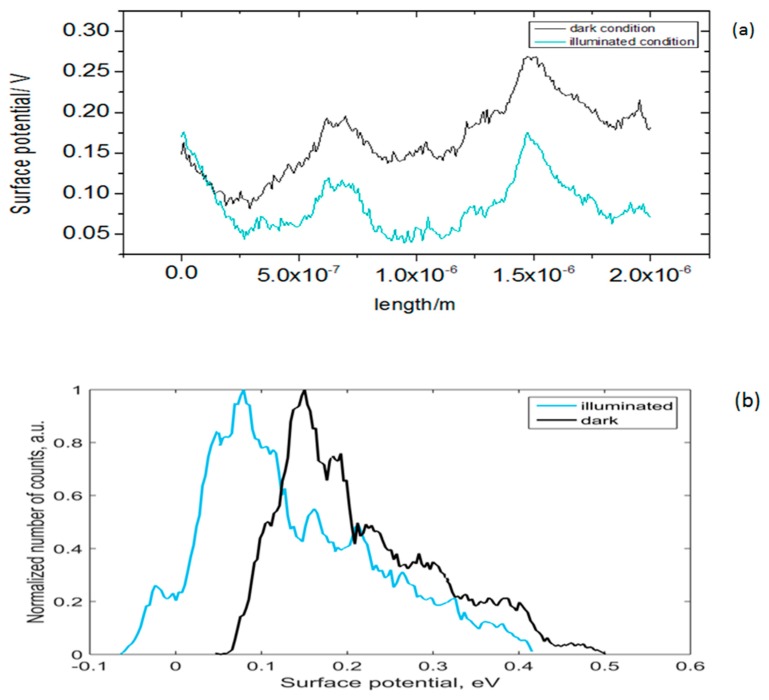
Graph of (**a**) the V_CPD_ profiles of the as-etched silicon nanoflakes under dark and illumination conditions and (**b**) V_CPD_ distribution.

The topography and surface potential measurements of silicon nanoflakes are presented in Figure 12 and Figure 13. As already shown in the SEM microstructural images and FIB sections, there was a significant variation in the thickness of the silicon nanoflakes formed. These were further confirmed by AFM topography (Figure 12a). Such variation may however, have an effect on the surface potential measurements due to topography crosstalk. In Figure 12b,c, V_CPD_ mapping of the silicon nanoflakes under both dark and illuminated conditions are shown. The V_CPD_ cross-section profiles of the silicon nanoflakes under dark and illuminated conditions are shown in Figure 13a and the V_CPD_ distribution in Figure 13b. Surface potentials measured under the illuminated condition were lower than under the dark condition.

## 4. Discussion

As the thickness of wafer based solar cells keeps reducing to decrease the materials cost-to-performance ratio, techniques requiring the removal of the top surface of the materials become inappropriate for surface modifications. For this reason, an easy fabrication route leading the creation if a nanostructured silicon surface will be desirable. The in situ formation of a nanostructured silicon coating on a wafer based solar cell presents an alternative method to produce textured wafer surfaces. Although preliminary results show the possibility to form silicon nanostructures that are well bonded to the wafer surface from FIB-SEM images in Figure 8, a number of synthesis parameters need to be improved. The thickness of the nanoflakes could not be controlled in this particular set-up. For efficient light reflection, scattering and absorption, a nanostructured coating of uniform thickness is desirable. The silicon nanoflakes showed a considerable decrease in surface reflection below 15% in the infrared region, compared to typical surface reflection of 45% for silicon wafer surface as shown in Figure 11. In the case of BSi, uniform peaks and valleys are created by removing portions of the top layer of the wafer surface. The thickness of the textured layer is easily controlled through process parameters as the layer forms a part of the bulk material. Very thin and uniformly distributed coating of the reaction slurry may be the key to controlling the thickness of the silicon nanoflakes. The morphology of this silicon type, although with peaks and valleys separated by micropores and cavities, is not easily controlled. 

Diatomite powder remains a suitable silica source due to the neatly arranged nanoporous structure [39]. Forming a thin silicon layer with retained nanoporous structures from precursor diatomite would be beneficial. Taking into account, the precursor diatomite powder was mechanically milled and calcined at 900 ˚C, an almost damaged diatomite powder was used in this experiment. In order to produce very porous silicon nanoflakes, it will be useful to colonize wafer surfaces with diatom frustules, followed by the MRR. This way a well structure precursor diatomite can be assured. The surface properties of the wafer play an important role in adhesion to this nanostructured coating. In this study, ethanol was used because it is known to improve the adhesive property of wafer surfaces. Cluster of silicon nanoflakes suggest however, that further surface treatments need to been done to improve the overall uniformity of the flakes as shown in Figure 5c. It would be desirable to cover the entire surface of the wafer to make way for commercial use.

Although this approach remains more ecofriendly compared to some BSi techniques requiring the use of *SF*_6_, the use of HF to etch unreacted silica remains a concern. Synthesis parameters that can help avoid the use of HF will make this approach the easiest fabrication route to obtain nanostructured silicon on the wafer surface. In almost all the techniques involving the fabrication of porous silicon, HF is predominant. The right mixing ratio for SiO_2_/Mg may be a crucial tool to achieve this. While the right mixing ratio of SiO_2_/Mg may lead to being more user friendly, an increase in Mg contamination also poses a concern. Magnesium contamination can however be easily resolved through HCl washing. Byproducts such as MgO and unreacted Mg will easily dissolve in HCl. The use of Raman spectroscopy provides a means to analyze the purity of silicon structures. From Raman spectroscopy results, shown in Figure 10, the first and second order scattering of silicon are observed. The first and second order Raman spectra are attributed to the main one-phonon peak and two-phonon overtone of the Γ-point optical phonon as reported by Uchinokura et al. [40]. Additionally, the difference in the FWHM values for the silicon wafer and the silicon nanoflakes can be related to the level of disorder in the different silicon types. Increasing FWHM has been attributed to increasing disorder in materials. The polycrystalline and/or amorphous nature of the silicon nanoflakes may account for this. In the substrate however, it is expected that a single crystal will have a low level of disorder, and hence a smaller FWHM value for the silicon wafer substrate. The formation of high purity porous silicon nanoflakes will make further surface texturing techniques appropriate without issues of size constraints. Herein, the surface of the bulk silicon will not be textured but the formed porous silicon coating, should there be a need to modify the structure.

The possibility to create an n-type silicon coating on a p-type silicon wafer or vice versa in a single step process is desirable. This provides a means to form a p–n junction. For this reason, it is necessary to know the doping type of the silicon nanoflakes formed. As already seen in FIB milling images in Figure 8 and an adhesion scratch test in Figure 9, a good bonding between the wafer surface and the silicon nanoflakes means a p–n junction would form automatically if fine chemistry tuning is applied during the MRR process. The formation of a depletion region in a p–n junction is known to suppress the recombination of electrons and holes generated upon excitation with photons from a light source. Measured surface potential values are related with the work function of materials, which also relates directly to the doping type of a semiconductor. Under super band gap illumination, band bending of the depleted surface decreases, which may be detected by a shift of surface photovoltage. This shift is opposite for n- and p-type semiconductors. Although, our results (Figure 12 and Figure 13) might suggest that the silicon nanoflakes were n-type, the dopant in this case was not easily identified. Food grade diatomite powders (such as used in this research) are known to contain trace elements of phosphorus [41] at concentration below the detection limit of the EDS analysis. The diffusion of a dopant from the substrate into the nanoflakes is also a route for doping of in situ synthesized nanoporous silicon. 

## 5. Conclusions

In summary, nanoporous silicon was successfully synthesized from diatomite powder via the magnesiothermic reduction reaction. Silicon nanoflakes formed directly on the silicon wafer possessed very intricate details of the precursor diatomite powder to some extent. The as-produced silicon nanoflakes reduced surface reflection of silicon wafer surface to values below 15%, reported a range of reflectivity for BSi. Although metal contamination from Mg in the form of Mg_2_Si, are possible, a follow-up self-etching of this unwanted product could also occur through the solid state reduction of SiO_2_. This can increase the silicon yield and self-control the thermite reaction. The high purity Si nanoflakes formed via the *in situ* MRR of diatomite was confirmed by results from EDS and Raman spectroscopy. A preliminary adhesion scratch test also confirmed a good bonding of the silicon nanoflakes to the surface of the substrate silicon wafer. We present a new strategy to produce nanostructured silicon wafer surfaces. Despite difficulties controlling the thickness and the uniformity of the nanostructured silicon formed, this technique is very promising, considering the ready availability, low cost, and ecofriendly nature of all starting materials, magnesium, diatomite, and sodium chloride, and byproducts. Additionally, this approach benefits directly from nature’s nanoporous structure present in the frustules of diatom algae, so that the current approach both mimics and draws inspiration from nature. This approach and materials offers an alternative route to the nanostructuring of silicon wafer surfaces for functional applications.

## Figures and Tables

**Table 1:** Chemical composition of diatomite powder.

Component.	SiO_2_	Al_2_O_3_	Fe_2_O_3_	MgO	K_2_O	Na_2_O
Quantity (wt%)	86%	5.5%	2.5%	0.78%	1.39%	0.22%
